# Excitatory Amino Acid Receptors Mediate Asymmetry and Lateralization in the Descending Cardiovascular Pathways from the Dorsomedial Hypothalamus

**DOI:** 10.1371/journal.pone.0112412

**Published:** 2014-11-14

**Authors:** Carlos Henrique Xavier, Danielle Ianzer, Augusto Martins Lima, Fernanda Ribeiro Marins, Gustavo Rodrigues Pedrino, Gisele Vaz, Gustavo Batista Menezes, Eugene Nalivaiko, Marco Antônio Peliky Fontes

**Affiliations:** 1 Laboratório de Fisiologia e Terapêutica Cardiovascular, Departamento Ciências Fisiológicas, Instituto de Ciências Biológicas, Universidade Federal de Goiás, Goiânia, GO, Brazil; 2 Departamento de Fisiologia e Biofísica, Instituto de Ciências Biológicas, Universidade Federal de Minas Gerais, Belo Horizonte, MG, Brazil; 3 School of Biomedical Sciences and Pharmacy, University of Newcastle, Newcastle, NSW, Australia; 4 Departamento de Bioquímica e Imunologia, Instituto de Ciências Biológicas, Universidade Federal de Minas Gerais, Belo Horizonte, MG, Brazil; University of Medicine & Dentistry of NJ - New Jersey Medical School, United States of America

## Abstract

The dorsomedial hypothalamus (DMH) and lateral/dorsolateral periaqueductal gray (PAG) are anatomically and functionally connected. Both the DMH and PAG depend on glutamatergic inputs for activation. We recently reported that removal of GABA-ergic tone in the unilateral DMH produces: *asymmetry*, that is, a right- (R-) sided predominance in cardiac chronotropism, and *lateralization*, that is, a greater increase in ipsilateral renal sympathetic activity (RSNA). In the current study, we investigated whether excitatory amino acid (EAA) receptors in the DMH–PAG pathway contribute to the functional interhemispheric difference. In urethane (1.2 to 1.4 g/kg, i.p.) anesthetized rats, we observed that: (i) nanoinjections of N-methyl D-aspartate (NMDA 100 pmol/100 nl) into the unilateral DMH produced the same right-sided predominance in the control of cardiac chronotropy, (ii) nanoinjections of NMDA into the ipsilateral DMH or PAG evoked lateralized RSNA responses, and (iii) blockade of EAA receptors in the unilateral DMH attenuated the cardiovascular responses evoked by injection of NMDA into either the R- or left- (L-) PAG. In awake rats, nanoinjection of kynurenic acid (1 nmol/100 nL) into the L-DMH or R- or L-PAG attenuated the tachycardia evoked by air stress. However, the magnitude of stress-evoked tachycardia was smallest when the EAA receptors of the R-DMH were blocked. We conclude that EAA receptors contribute to the right-sided predominance in cardiac chronotropism. This interhemispheric difference that involves EAA receptors was observed in the DMH but not in the PAG.

## Introduction

Asymmetries are the left-right differences in the properties of central nervous system and are found at different levels of the neuraxis [1], [2]. There is evidence for interhemispheric specialization of autonomic control in humans [3], [4]. During stress and emotional arousal, most brain regions are influenced asymmetrically, which unbalances sympathetic outflow to the heart and improves the chances of cardiac ectopies [5], [6].

Studies have highlighted the involvement of diencephalic and mesencephalic regions in the organization of physiological responses to stress [for review, see [7], [8]]. Anatomic and functional data have clearly revealed that the hypothalamus and the lateral/dorsolateral regions of the periaqueductal gray (PAG) are connected [9]–[17].

The dorsomedial hypothalamus (DMH) region plays a key role in organizing physiological responses to emotional stress [18]. DMH neurons are excited by nanoinjections of either GABA_A_ antagonists or excitatory amino acid (EAA) agonists [8]. The inhibition of DMH neurons attenuates the responses evoked by exposure to emotional stress [19]. Inversely, DMH disinhibition via the blockade of local GABA_A_ receptors produces a series of effects that mimic stress-related physiological responses, such as behavioral changes, thermogenesis, tachycardia and the pressor response (for review, see [8]). Previous studies have demonstrated that this selective GABA-ergic blockade results in DMH activation that allows for a predominant local excitatory input [20], [21]. These findings suggest that both phenomena should happen simultaneously to produce the aforementioned responses.

The PAG is thought to be an exit synaptic relay for defensive reactions [22], [23]. Similar to the DMH, the PAG also mediates physiological responses to stress [7], [24]. The PAG is responsive to efferent excitatory inputs [25] from higher regions [26], such as the DMH [12]. Activation of the PAG with the excitatory amino acid (EAA) agonist N-methyl D-aspartate (NMDA), especially in the lateral and dorsolateral columns, evokes behavioral changes and sympathetically mediated increases in body temperature, heart rate (HR) and arterial pressure [12], [13]. Recent studies have revealed a functional projection between the DMH and the PAG [12], [13], [27]. The tachycardia evoked by the unilateral DMH depends on EAA receptors in the PAG, particularly NMDA receptors [12]. In addition to the fact that DMH-evoked responses relays in PAG [8], the activity of DMH neurons is similarly required for PAG-evoked responses [13], [27]. In this regard, DMH-PAG connections seem to be a bilateral excitatory pathway that allows setting specific responses.

We have previously reported that the cardiovascular responses evoked by nanoinjections of a GABA_A_ antagonist into unilateral (left or right) DMH have the following characteristics: i) asymmetry – the right DMH drives full cardiac performance (chronotropy and inotropy) and tail vasoconstriction; and ii) lateralization – either side of the DMH seems to prominently exert autonomic control over the ipsilateral hemibody [18], [28], [29]. Thus, the question of whether excitatory input contributes to the asymmetry and lateralization at the level of the DMH and PAG arises. In the present study, we compared the cardiovascular responses evoked by activation and blockade of EAA receptors in the right and left sides of the DMH and PAG. A portion of our results have been presented in abstract form [30].

## Results

Photomicrographs of coronal sections of the DMH and PAG exemplifying injection sites targeted in our experiments are, such as an schematic drawing on the injections sites obtained in all experiments are shown in Figure S1. The baseline values that were obtained before the initiation of the experiments are shown in table 1. There were no differences in the baseline values that were sampled prior to the administration of the central nanoinjections of each experimental series.

**Table 1:** Baseline values of mean arterial pressure (MAP) and heart rate (HR) collected before nanoinjections into unilateral DMH or PAG.

	Anesthetized				Non-Anesthetized			
	NMDA DMH		NMDA PAG		Kyn DMH		Kyn PAG	
	Left	Right	Left	Right	Left	Right	Left	Right
MAP (mmHg)	93±3	94±2	96±6	98±7	102±3	105±5	106±4	108±8
HR (bpm)	319±13	325±4	353±9	357±10	321±8	334±9	346±13	333±9

There were no differences between basal values found before nanoinjections between L- and R- within each experimental series.

### Experiment 1. Unilateral activation of the DMH and PAG increased heart rate, blood pressure and renal sympathetic activity

**Figure 1:**
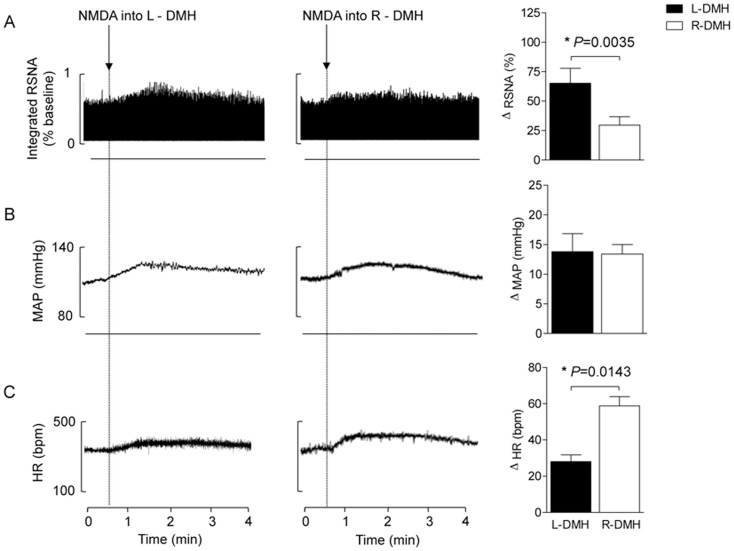
Representative chart records and changes in RSNA (A), MAP (B) and HR (C) evoked by the nanoinjection of NMDA (100 pmol/100 nl) into the right (white bars) and left (black bars) DMH in the first experimental series (n = 5). *P<0.05 Right- *vs.* Left-DMH (Mann Whitney non-parametric test).

**Figure 2:**
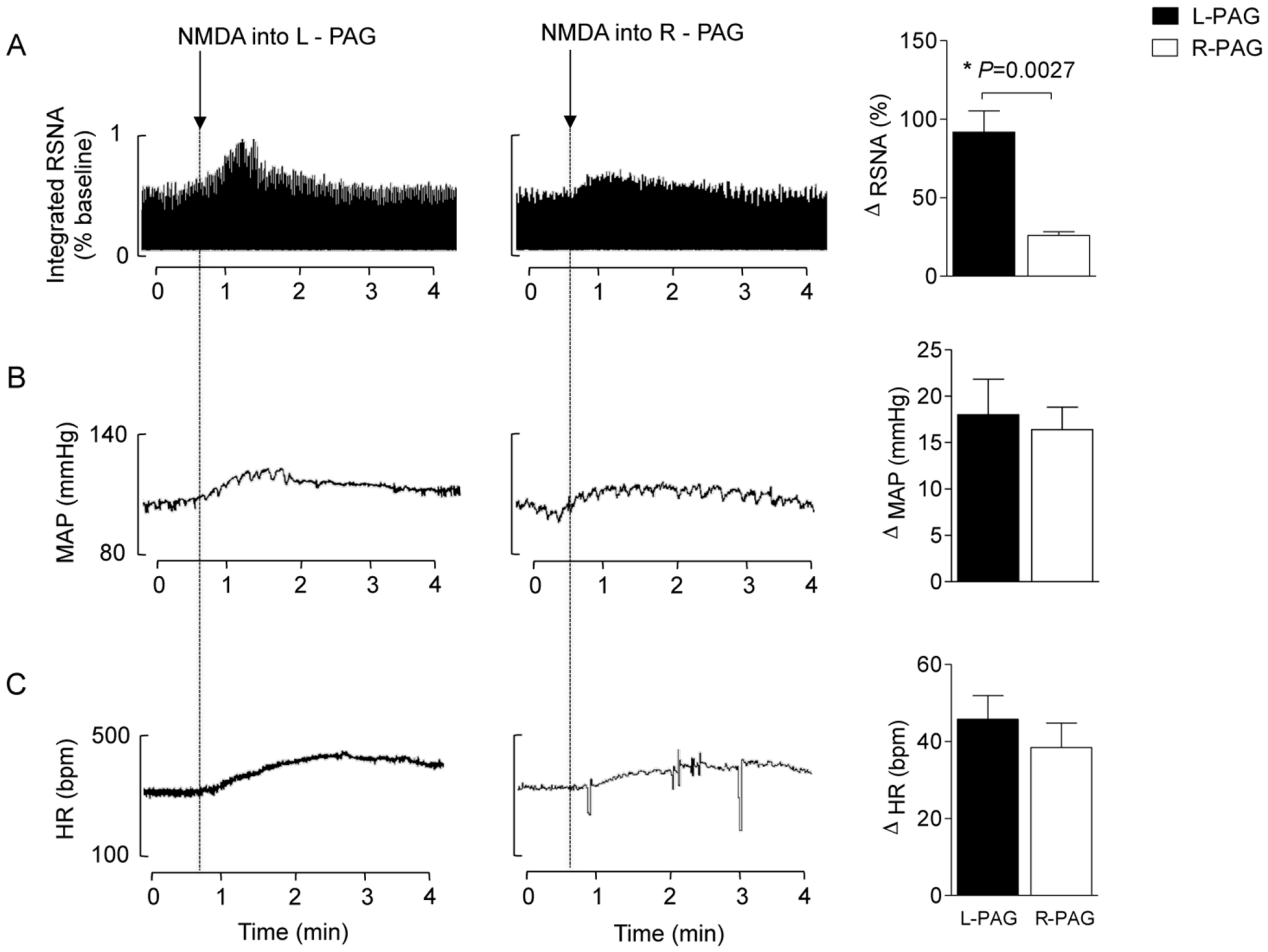
Representative chart records and changes in RSNA (A) (recorded in the left renal nerve), MAP (B) and HR (C) evoked by nanoinjections of NMDA (100 pmol/100 nl) into the right (white bars) and left (black bars) PAG in the first experimental series (n = 5). *P<0.05 Right- vs. Left-PAG (Mann Whitney non-parametric test).

**Figure 3:**
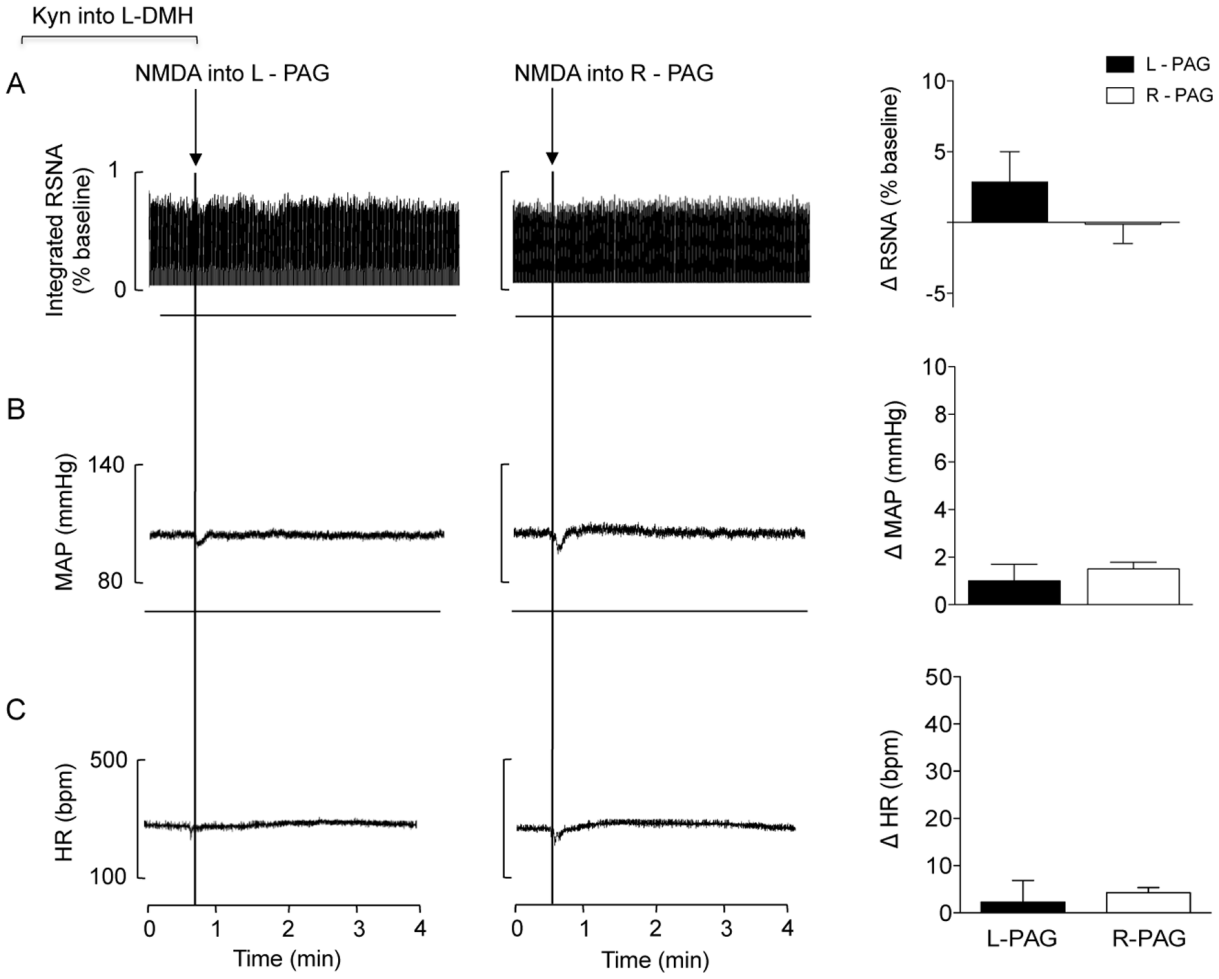
Representative chart records and changes in RSNA (A) (recorded in the left renal nerve), MAP (B) and HR (C) evoked by nanoinjections of NMDA (100 pmol/100 nl) into the right (white bars) and left (black bars) PAG after blockade of EAA receptors in the left DMH in the second experimental series (n = 5; Mann Whitney non-parametric test).

The results obtained and typical examples are grouped in figures 1 and 2. Nanoinjection of NMDA into the unilateral DMH (Figure 1) and PAG (Figure 2) positively affected autonomic and cardiovascular parameters. These effects were evident one minute after the nanoinjections and continued for approximately 5 minutes. We observed mean arterial pressure (MAP) increases after unilateral injections of NMDA into the DMH or PAG (Figures 1B and 2B; *P*<0.05). However no differences were found in the ranges of these responses when nanoinjections into the left or right side within the same experimental group were compared (ΔMAP DMH: Right  = 13±2 *vs.* Left side  = 14±3; PAG: Right  = 16±3 *vs.* Left side  = 18±4 mmHg; Figures 2B and 3B).

Although unilateral nanoinjections of NMDA into either side of the DMH or PAG increased RSNA as sampled in the left renal nerve, greater responses were found after injections into the left DMH and PAG; i.e., greater responses were observed after injections that were ipsilateral to the side in which RSNA was sampled (ΔRSNA DMH: Right  = 30±7 *vs.* Left side  = 66±13; PAG: Right  = 26±3 *vs.* Left side  = 92±14%; *P*<0.05; Figures 1A and 2A). Nanoinjections into the DMH and PAG also increased HR (*P*<0.05 vs. baseline; Figures 1C and 2C), but injections into the Right-DMH provoked greater chronotropy effect (ΔHR DMH: Right  = 59±5 *vs.* Left side  = 28±4 bpm; *P*<0.05; Figure 1C). The tachycardia evoked by nanoinjections of NMDA into the ipsilateral PAG was similar to that observed after injections into the contralateral side (ΔHR PAG: Right  = 39±6 *vs.* Left side  = 45±6 bpm; Figure 2C).

### Experiment 2. EAA receptors in the unilateral DMH contributed to the HR, MAP and RSNA responses evoked from the PAG

In contrast to the observations of experiment 1, blockade of the EAA receptors in the left DMH completely abolished the increases in RSNA, MAP and HR that were evoked by injections of NMDA into either the ipsilateral or contralateral PAG (ΔRSNA: Right  = −1±2 *vs.* Left side  = 3±5%; ΔMAP: Right  = 1±2 *vs.* Left side  = 2±1 mmHg; and ΔHR: Right  = 3±5 *vs.* Left side  = 4±1 bpm, Figure 3). We found no differences in asymmetry or lateralization in the control of HR or RSNA.

### Experiment 3. EAA receptors in the unilateral DMH and PAG are involved in stress-induced tachycardic and pressor responses in non-anaesthetized rats

**Figure 4:**
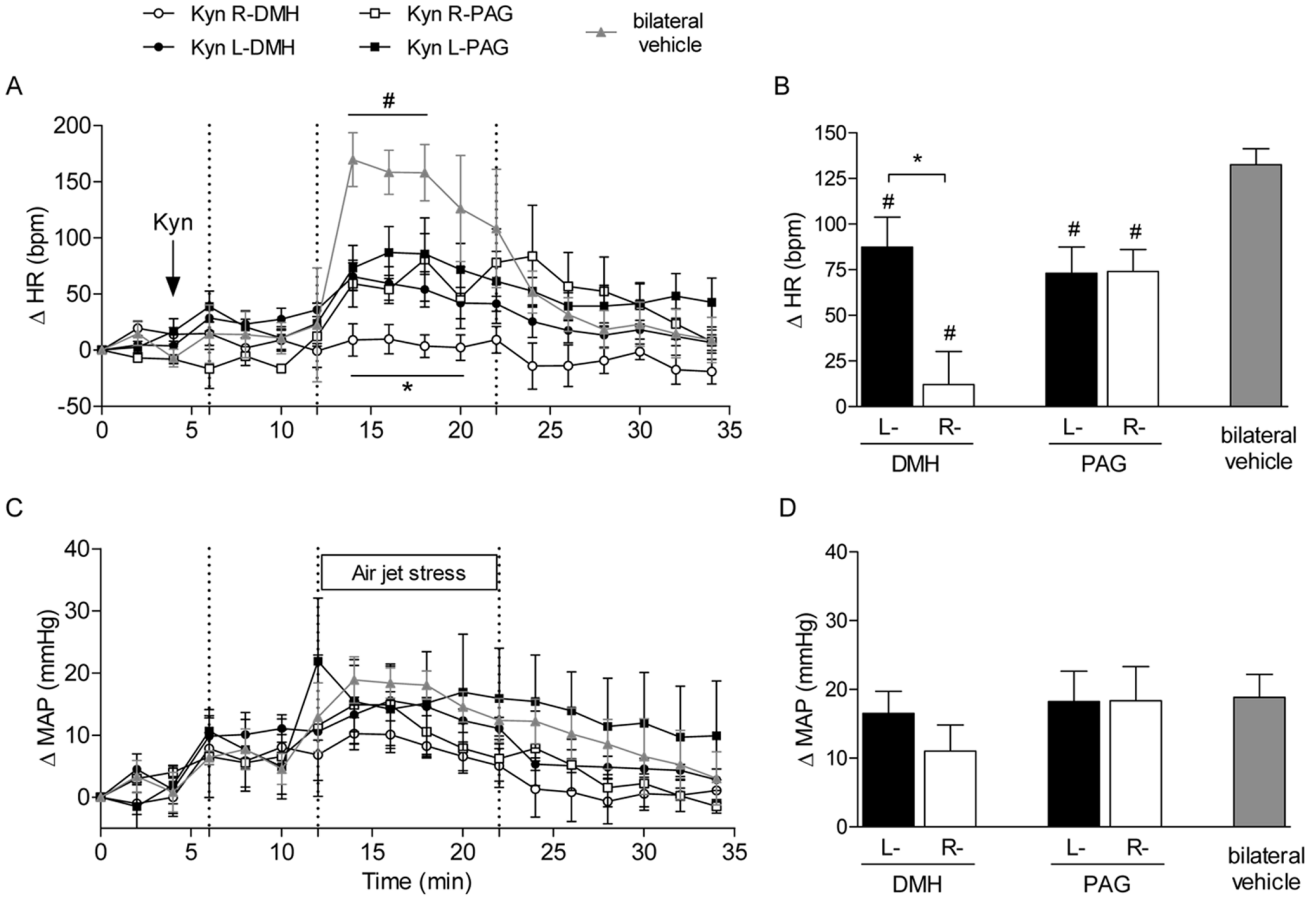
Results of the third experimental series. **Panels A and C**: time courses of HR and MAP before and after nanoinjection of kynurenic acid (1 nmol/100 nl) into the right (open circles) and left (black circles) DMH (n = 5) and right (open squares) and left (black squares) l/dl PAG (n = 5) and after bilateral vehicle nanoinjection (gray triangles; n = 10). *P<0.05 Right (R-) *vs.* Left (L-) DMH; ^#^ *P*<0.05 vehicle compared to R- or L-DMH and R- or L-PAG (two-way ANOVA followed by Newman Keuls post hoc tests). **Panels B and D**: peak changes in HR and MAP during the stress trials in animals that had previously been nanoinjected with kynurenic acid (1 nmol/100 nl) into right (open bars) and left (black bars) DMH or l/dl PAG and after the bilateral nanoinjection of vehicle (gray bars). *P<0.05 R *vs.* L and ^#^ *P*<0.05 *vs.* bilateral vehicle (one-way ANOVA followed by Newman-Keuls post hoc tests).

Figure 4 (panels A and C) shows the time courses of the responses over the entire durations of the experiments. Panels B and D show the peak changes in HR and MAP that were observed during stress exposure. Compared to the amplitudes of the control responses (bilateral vehicle bars – Figure 4B), nanoinjections of kynurenic acid into either the right or left-sides of the DMH or PAG attenuated the tachycardia evoked by stress. However, stress-evoked tachycardia was dramatically reduced when the EAA receptors in the Right-DMH were blocked (ΔHR DMH: Right  = 12±18 *vs.* Left side  = 87±17; PAG: Right  = 73±15 *vs.* Left side  = 74±12; bilateral vehicle: 132±9 bpm; *P*<0.05 – Figure 4B). Blockade of the EAA receptors in either the Right or Left-sides of the DMH and PAG did not alter the amplitudes of the pressor responses induced by stress (ΔMAP DMH: Right  = 12±4 *vs.* Left side  = 17±3; PAG: Right  = 18±4 *vs.* Left side  = 18±5; bilateral vehicle: 19±3 mmHg – Figure 4D).

### Experiment 4. Anatomic pathways between the DMH and PAG

**Figure 5:**
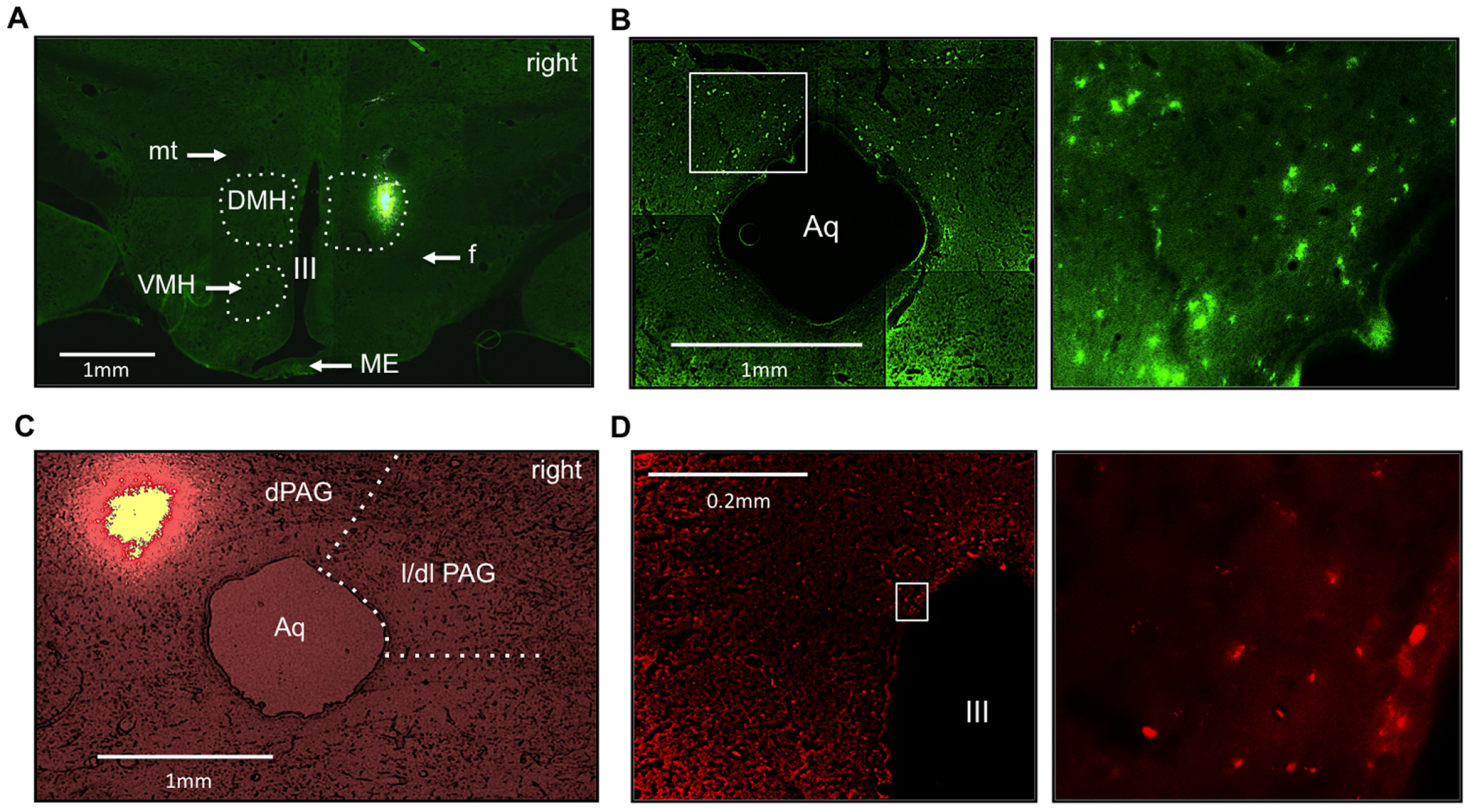
Photomicrographs of rat brain slices depicting spots from animals injected with green and red retrobeads at the level of (A) the DMH and (C) the PAG, respectively. Zoom view of the (B) PAG and (D) DMH neurons that were retrogradely labeled with green and red retrobeads, respectively. DMH: dorsomedial hypothalamus; ME: median eminence; mt: mammillothalamic tract; f: fornix; VMH: ventromedial hypothalamus; III: third ventricle; l/dlPAG: lateral/dorsolateral periaqueductal gray; Aq: aqueduct.

Photomicrographs depicting the injection sites of retrobeads into the right DMH and left PAG and labeled neurons are shown in Figure 5A and B. In the left DMH, neurons labeled with red beads that were originally injected into the left PAG were observed. Similarly, in the right PAG, neurons labeled green with beads injected into the right DMH were observed.

## Discussion

The major findings of this study are as follows: i) NMDA nanoinjected into the Right-DMH evoked greater cardiac chronotropic changes than did nanoinjections into the Left-DMH; ii) nanoinjection of NMDA into the unilateral DMH and PAG evoked lateralized increases in RSNA; and iii) blockade of EAA receptors in the right DMH nearly abolished stress-evoked tachycardia, whereas identical blockade in the Left-DMH, Left- and Right-PAG only attenuated the tachycardic response.

The present data, obtained in rats, extend previous findings showing that the right hypothalamus governs full cardiac performance [28], [29], [31]. We recently demonstrated that disinhibition of the Right-DMH produces greater tachycardia compared to disinhibition of the Left-DMH. Additionally, inhibition of the Right-DMH nearly abolished stress-evoked tachycardia [28]. The removal of GABA-ergic tone (disinhibition) implicates in predominant local excitatory input to the DMH, mainly by recruiting inotropic glutamate receptors [20], [21], which changes the sympathetic output [8] and regional blood flow to different target organs [28], [32]. The present study demonstrates that this asymmetric cardiac control by GABA_A_ receptors also involves EAA receptors in the DMH. Together with the greater positive chronotropy that is evoked by NMDA injections into Right-DMH, the lack of cardiac reactivity during the stress trial under the blockade of EAA receptors in the right side highlights the involvement of excitatory input in DMH asymmetry. Whether these differential cardiac responses are governed by the amygdala [33] or other sources of excitatory input to the DMH [34] remains to be revealed.

Newly described findings suggest that the greater chronotropic response that is evoked by removal of GABA-ergic tone in the Right-DMH [28] may involve differential glutamatergic inputs. Several mechanisms could account for this difference. One possibility is that this functional asymmetry results from an unknown characteristic of the DMH neurons in the right side, which may be determined by differences in electrophysiological properties or the densities of glutamatergic receptors. It is also important to consider other possible functional and neuroanatomical differences between the right and left pathways that descend from the DMH, including relays other than the PAG, and the organization of sympathetic projections to the heart. Because the DMH projects and provides glutamatergic outputs to other nuclei that are involved in the control of cardiovascular responses, such as rostral ventrolateral medulla (RVLM) [35] and raphe pallidus (RP) [36], it is plausible that these lower relays are involved. Additionally, the right and left cardiac sympathetic neurons and nerves differ in terms of function [37]–[40], and the stimulation of the components on the right side is more likely to evoke positive chronotropic responses [39], [41], [42].

We found that nanoinjection of NMDA into the unilateral DMH or PAG evoked greater responses in the ipsilateral renal nerve. This finding indicates that the control of RSNA by the DMH and PAG is lateralized. For technical reasons, RSNA was recorded in the left nerve. We recognize that simultaneous recordings of both nerves would be more appropriate; however, in a previous study, we also observed that blockade of GABA_A_ receptors in the DMH also evokes greater increases in the ipsilateral nerve [28]. Therefore, the current data together with data from our previous study support our hypothesis of a certain degree of hypothalamic lateralization in the control of RSNA [28]. In fact, substantial evidences in the literature support the existence of lateralization in the control of RSNA and other autonomic nerves [43]–[46]. Previous anatomical studies further support this idea of lateralization. In this regard, the descending pathways from the DMH are organized as ipsilateral mirrors [9], [47]. Accordingly, Zaretskaia and colleagues clearly demonstrated the recruitment of predominantly ipsilateral relays after DMH disinhibition [48]. However, the pattern of lateralization in the autonomic control of renal nerves by PAG differed from that observed after exteroceptive stimulation in conscious rats [23]. Also, it has been reported that unilateral nanoinjection of a nitric oxide donor into the PAG recruits ipsilateral DMH neurons, which suggests that the PAG-DMH pathway underlies organized responses that may not be the consequence of generalized arousal [49]. Because the PAG is one of the descending pathways from the DMH [8], [18] and the activity of DMH neurons is required for PAG-evoked responses [13], [27], we suggest that the functional lateralization between the DMH and PAG composes a collateral background facilitation system for controlling RSNA. Our anatomic findings extend this idea of DMH–PAG bidirectional connection.

Inhibition of the PAG attenuated stress-induced tachycardia, as previously described [24]. In our experiments, we observed that there was no asymmetry in the cardiac control by PAG, which may indicate that EAA receptors in both sides of PAG participate equally in cardiac reactivity to stress. Also, the blockade of EAA receptors in the Left-DMH attenuated the cardiovascular responses evoked by either ipsilateral or contralateral PAG stimulation. Because the right DMH remains functioning after the blockade of the contralateral DMH, we suggest that the PAG-DMH pathway may be involved in the organization of lateralized but not asymmetric responses. Additionally, unilateral PAG stimulation failed to reveal any cardiac dominance, which indicates that the excitatory input from the PAG is not involved in asymmetric cardiac control – as observed for the DMH. We also confirmed that the PAG indeed acts via the DMH [13], [27]. However, an important question requires further investigation; i.e., whether the descending pathways from the DMH are the pathways that govern the PAG-evoked responses.

A growing body of research has reported on the interactions between the DMH and PAG in the organization of cardiovascular responses to emotional stress. Our recent results add the contribution of EAA receptors in the DMH-PAG pathway to the evidence suggesting that interhemispheric differences are capable of modifying cardiovascular control. Undoubtedly, further studies are necessary to better understand the pathways and mechanisms underlying this functional interhemispheric difference. Studying the differential autonomic responses that are organized by each side of the brain may improve the knowledge about the interindividual variability of stress-evoked responses.

## Methods

### 4. 1 General procedures

All experiments were performed on male Wistar rats (250–320 g; 10–12 weeks) that were bred at the animal facilities of the Biological Sciences Institute (CEBIO, UFMG, Belo Horizonte, MG, Brazil) and were conducted in accordance with the guidelines established by our local committee and in accordance with the U.S. National Institutes of Health Guide for the Care and Use of Laboratory Animals. This study was approved by the institutional animal care and use committee – (Comitê de Ética em Experimentação Animal - CETEA/UFMG protocol number 137/06). All efforts were made to minimize the number of animals used. Animals were housed in individual home cages (47 cm×31×cm×16 cm) and had free access to food and water.

We recorded the following cardiovascular parameters in two different experimental conditions: *i)* in the *anesthetized* condition, we recorded HR, MAP and renal sympathetic nerve activity (RSNA); and ii) in the *non-anesthetized* condition, we recorded HR and MAP.

### 4.2 Experiments in anesthetized animals

#### General procedures

Rats were anesthetized with urethane (1.2 to 1.4 g/kg i.p.). The trachea was cannulated to maintain an open airway. The adequacy of the anesthesia was verified by the absence of a withdrawal response to nociceptive stimulation of the hindpaw. Supplemental doses of urethane (0.1 g/kg i.v.) were given when necessary. Body temperature was maintained near 37.0°C with a heating lamp. The head was positioned in a stereotaxic frame (Stoelting, Wood Dale, IL, USA) with the tooth bar fixed at −3.3 mm below the interaural line. Using a low rotation drill, small craniotomies were made for the insertion of thin-tip graduated glass pipettes into the DMH (3.2 mm posterior, 0.6 mm lateral and 8.5 mm ventral) and the PAG (7.0 mm posterior, 0.6 mm lateral and 4.8 mm ventral).

Catheters were placed into a femoral artery to record MAP and HR and into a femoral vein for supplementary anesthesia. The left renal nerve and left renal artery were isolated and covered with mineral oil. Subsequently, the renal nerve was placed on a silver bipolar electrode. The RSNA signal was amplified (10K), filtered (100–1000 Hz), displayed on an oscilloscope and monitored by via an audio amplifier. The filtered nerve activity signal was rectified, integrated (resetting every second), displayed online and acquired using Powerlab 4/20 LabChart 7.1 (ADInstruments, Sydney, Australia). All data were digitized at 1 kHz. The noise of the recording system was determined *post mortem* and subtracted from the RSNA values obtained during the experiment. After a 20-minutes stabilization period, the experiments were performed as described below.

#### Experiment 1. Effects of unilateral stimulation of the DMH and PAG on HR, MAP and RSNA

We evaluated the changes in HR, MAP and RSNA that were evoked by nanoinjection of NMDA (100 pmol/100 nl) into the left or right DMH and PAG. These experiments were performed in two separate groups of animals (n = 5 each) that received nanoinjections into the DMH or PAG. The side of the first unilateral DMH or PAG injection was chosen randomly. If NMDA was injected into the R-DMH, the subsequent injection was into the L-DMH. The same strategy was adopted for nanoinjections into the PAG. Between nanoinjections, we waited a minimum of 15 minutes or until the variables returned to baseline.

#### Experiment 2. Contribution of the EAA receptors of the unilateral DMH to the PAG-evoked responses

Based on previous reports that examined the influence of DMH neurons on the responses evoked from the PAG [13], [27], we aimed to reveal whether blockade of EAA receptors in the unilateral DMH could modify the range of the responses evoked by stimulation of either the unilateral or contralateral PAG (n = 5). Following a 20-minutes stabilization period, we nanoinjected kynurenic Acid (Kyn, an antagonist of ionotropic AMPA, NMDA and kainate glutamate receptors; 1 nmol/100 nl) into the left DMH. After 15 minutes, we performed the first injection of NMDA (100 pmol/100 nl) into the unilateral PAG (the side was randomly chosen). Fifteen minutes later or when the variables returned to baseline, the second nanoinjection was made into the contralateral PAG.

### 4.3 Experiments in non-anesthetized animals

#### General procedures

Under tribromoethanol anesthesia (250 mg/kg i.p.), the rats were placed in a stereotaxic frame (Stoelting, Wood Dale, IL, USA) and bilateral stainless steel guide cannulas (22 gauge, 16 mm length) targeted toward the DMH or PAG were inserted as previously described [28]. After the surgical procedures, the animals were allowed to recover in individual home cages for at least three days. Subsequently, the animals were anesthetized again with tribromoethanol (250 mg/kg i.p.), and a polyethylene catheter (0.011 ID, Clay Adams, Parsippany, NJ, USA) was inserted into a femoral artery to record MAP and HR. The catheter was routed subcutaneously to the nape of the neck where it was exteriorized and secured. Rats were then allowed to recover for 24-h before the experiments began. All animals for which data were reported remained in good health conditions throughout the course of surgical procedures and experimental protocol as assessed by appearance, behavior, and maintenance of body weight.

#### Experiment 3. Contributions of EAA receptors of the unilateral DMH and PAG to the stress-induced tachycardic and pressor responses

We assessed the contributions of EAA receptors in the unilateral DMH or PAG to the cardiovascular responses evoked by acute stress exposure. In one group of animals (n = 5), bilateral guide cannulas were targeted only to the DMH. In another group of animals (n = 5), guide cannulas were targeted only to the bilateral PAG (the coordinates are provided below). Both the DMH and PAG groups were subjected to the following experimental procedures. After 20 minutes of baseline HR and MAP monitoring, the EAA antagonist Kyn (1 nmol/100 nl) was injected unilaterally into the DMH or PAG. The rats were then placed into a plastic restrainer (60-mm inner diameter) and subjected to an air jet (10 l/min) directed toward their heads for 10 min. Subsequently, the animals remained in the restrainer for an additional 10 min. The air jet stress was repeated on 3 consecutive days. On the first day, the side of the first nanoinjection was chosen randomly. On the second day, the nanoinjection was contralateral to the site targeted on the first day. As a control, on the third day, the stress trial was repeated after bilateral nanoinjection of vehicle (NaCl 0.9%, 100 nl) in all animals. After the surgeries, a single doses of antibiotics [Pentabiotic (Benzathine Benzilpeniciline, Sodium Benzilpeniciline, Potassium Benzilpeniciline, Procaine Benzilpeniciline and Estreptomicin) 5 mg/kg; 0.2 ml] and analgesics [Banamine Pet (Flunixim Meglumine) 1.1 mg/kg] were injected (i.m.).

### 4.4 Anatomic tracings

#### Experiment 4. Anatomic pathways between the DMH and PAG

Anesthetic procedures were as described for Experiment 2. The craniotomy procedures used to access the DMH and PAG were identical to those used in Experiment 1. The procedures for the neuronal tracings were as previously described [50]. Undiluted green and red Retrobeads (Luma Fluor) were nanoinjected (100 nl) into the right DMH and left PAG, respectively. Seven days later, the brain were removed, maintained in 4% PFA for 24-h and then transferred to a 30% sucrose solution for 48 h. Brain slices (40 µm) containing the DMH and PAG regions were taken and mounted on silanized glass slides. Microscopic analyses were performed using excitation maximums of 460 and 530 nm and emission maximums of 505 and 590 nm for the green and red beads, respectively. Procedures for analgesia and antibiotic injections were the same described above (Experiment 3).

### 4.5 Injections and drugs

The following drugs were utilized in the physiological experiments: i) NMDA (100 pmol/100 nl); ii) the EAA receptor antagonist kynurenic acid (1 nmol/100 nl); and iii) vehicle (NaCl 0.9%, 100 nl). The drugs were purchased from Sigma. As control, some injections of vehicle (NaCl 0.9%) – 100 nL) were performed. The coordinates for the nanoinjections into the DMH were −3.2 mm posterior and ±0.6 mm lateral to bregma with a depth of −8.5 mm. The coordinates for the PAG nanoinjections were −7.0 mm posterior and ±0.6 mm lateral to bregma at a depth of −4.8 mm [51].

### 4.6 Histology

At the end of the experiments (1–3), alcian blue dye (2%, 100 nl) was nanoinjected into the DMH and PAG for subsequent histological confirmation [52], [53]. Subsequently, the rats were euthanized by overdoses of anesthetic. The atlas of Paxinos & Watson was used as reference to confirm the injection sites in the DMH and PAG [51].

### 4.7 Analyses

Comparisons between maximal changes (sampled before and after) evoked by nanoinjections into DMH or PAG were determined by Mann Whitney non-parametric test. In air jet experiments, comparisons of the stress-evoked changes between groups (sampled every 2 min) were performed with two-way analysis of variance (factors drug and time) followed by Newman Keuls post hoc test. Peak changes in HR and MAP induced by air jet were compared by one-way analysis of variance followed by Newman Keuls post hoc test. We used Mann Whitney test and ANOVAs as appropriate (see figure legends for details). The level of significance was taken as *P*<0.05. The data are expressed as the mean ±SEM.

## Supporting Information

Figure S1 **Photomicrographs of rat brain slices and schematic drawing depicting the injection sites at the level of the DMH (column A) and PAG (column B).** mt: mammillothalamic tract; f: fornix; III: third ventricle; l/dlPAG: lateral/dorsolateral periaqueductal gray; Aq: aqueduct. White squares: experiment 1. Black squares: experiment 2. White circles: experiment 3.
(TIF)

## References

[1] Harris JA, Guglielmotti V, Bentivoglio M (1996) Diencephalic asymmetries. Neurosci Biobehav Rev 20: 637–643. 8994203 10.1016/0149-7634(95)00077-1

[2] Toga AW, Thompson PM (2003) Mapping brain asymmetry. Nat Rev Neurosci 4: 37–48. 12511860 10.1038/nrn1009

[3] Oppenheimer S (2006) Cerebrogenic cardiac arrhythmias: cortical lateralization and clinical significance. Clin Auton Res 16: 6–11. 16477489 10.1007/s10286-006-0276-0 PMC2782122

[4] Lane RD, Critchley HD, Taggart P (2014) Asymmetric autonomic innervation. In Handbook of Cardiovascular Behavioral Medicine Edited by Waldstein S, Kop W and Katzel L New York, Springer. In press.

[5] Critchley HD, Taggart P, Sutton PM, Holdright DR, Batchvarov V, et al (2005) Mental stress and sudden cardiac death: asymmetric midbrain activity as a linking mechanism. Brain 128: 75–85. 15496434 10.1093/brain/awh324

[6] Wittling W (1997) The right hemisphere and the human stress response. Acta Physiol Scand Suppl 640: 55–59. 9401607

[7] Carrive P (2009) Emotional Control of the Autonomic Nervous System. In: Larry RS, editor.Encyclopedia of Neuroscience.Oxford: Academic Press. pp.923–928.

[8] Fontes MA, Xavier CH, de Menezes RC, Dimicco JA (2011) The dorsomedial hypothalamus and the central pathways involved in the cardiovascular response to emotional stress. Neuroscience 184: 64–74. 21435377 10.1016/j.neuroscience.2011.03.018

[9] ter Horst GJ, Luiten PG (1986) The projections of the dorsomedial hypothalamic nucleus in the rat. Brain Res Bull 16: 231–248. 3697791 10.1016/0361-9230(86)90038-9

[10] Farkas E, Jansen AS, Loewy AD (1998) Periaqueductal gray matter input to cardiac-related sympathetic premotor neurons. Brain Res 792: 179–192. 9593884 10.1016/s0006-8993(98)00029-8

[11] Carrive P, Gorissen M (2008) Premotor sympathetic neurons of conditioned fear in the rat. Eur J Neurosci 28: 428–446. 18702716 10.1111/j.1460-9568.2008.06351.x

[12] da Silva LG Jr, Menezes RC, Villela DC, Fontes MA (2006) Excitatory amino acid receptors in the periaqueductal gray mediate the cardiovascular response evoked by activation of dorsomedial hypothalamic neurons. Neuroscience 139: 1129–1139. 16458440 10.1016/j.neuroscience.2005.12.041

[13] de Menezes RC, Zaretsky DV, Fontes MA, DiMicco JA (2009) Cardiovascular and thermal responses evoked from the periaqueductal grey require neuronal activity in the hypothalamus. J Physiol 587: 1201–1215. 19171660 10.1113/jphysiol.2008.161463 PMC2674992

[14] Vianna DM, Brandao ML (2003) Anatomical connections of the periaqueductal gray: specific neural substrates for different kinds of fear. Braz J Med Biol Res 36: 557–566. 12715074 10.1590/s0100-879x2003000500002

[15] Bandler R, Keay KA (1996) Columnar organization in the midbrain periaqueductal gray and the integration of emotional expression. Prog Brain Res 107: 285–300. 8782526 10.1016/s0079-6123(08)61871-3

[16] Bernard JF, Bandler R (1998) Parallel circuits for emotional coping behaviour: new pieces in the puzzle. J Comp Neurol 401: 429–436. 9826271

[17] Kerman IA (2008) Organization of brain somatomotor-sympathetic circuits. Exp Brain Res 187: 1–16. 18369609 10.1007/s00221-008-1337-5

[18] Fontes MA, Xavier CH, Marins FR, Limborco-Filho M, Vaz GC, et al (2014) Emotional stress and sympathetic activity: Contribution of dorsomedial hypothalamus to cardiac arrhythmias. Brain Res 1554C: 49–58. 10.1016/j.brainres.2014.01.043 24491632

[19] Stotz-Potter EH, Willis LR, DiMicco JA (1996) Muscimol acts in dorsomedial but not paraventricular hypothalamic nucleus to suppress cardiovascular effects of stress. J Neurosci 16: 1173–1179. 8558246 10.1523/JNEUROSCI.16-03-01173.1996 PMC6578823

[20] Soltis RP, DiMicco JA (1991) Interaction of hypothalamic GABAA and excitatory amino acid receptors controlling heart rate in rats. Am J Physiol 261: R427–433. 1678933 10.1152/ajpregu.1991.261.2.R427

[21] Soltis RP, DiMicco JA (1991) GABAA and excitatory amino acid receptors in dorsomedial hypothalamus and heart rate in rats. Am J Physiol 260: R13–20. 1671541 10.1152/ajpregu.1991.260.1.R13

[22] Carrive P (1993) The periaqueductal gray and defensive behavior: functional representation and neuronal organization. Behav Brain Res 58: 27–47. 8136048 10.1016/0166-4328(93)90088-8

[23] Bandler R, Carrive P (1988) Integrated defence reaction elicited by excitatory amino acid microinjection in the midbrain periaqueductal grey region of the unrestrained cat. Brain Res 439: 95–106. 3359200 10.1016/0006-8993(88)91465-5

[24] de Menezes RC, Zaretsky DV, Sarkar S, Fontes MA, Dimicco JA (2008) Microinjection of muscimol into the periaqueductal gray suppresses cardiovascular and neuroendocrine response to air jet stress in conscious rats. Am J Physiol Regul Integr Comp Physiol 295: R881–890. 18650321 10.1152/ajpregu.00181.2008 PMC2536852

[25] Albin RL, Makowiec RL, Hollingsworth Z, Dure LS IV, Penney JB, et al (1990) Excitatory amino acid binding sites in the periaqueductal gray of the rat. Neurosci Lett 118: 112–115. 2175406 10.1016/0304-3940(90)90261-7

[26] Beart PM, Summers RJ, Stephenson JA, Cook CJ, Christie MJ (1990) Excitatory amino acid projections to the periaqueductal gray in the rat: a retrograde transport study utilizing D[3H]aspartate and [3H]GABA. Neuroscience 34: 163–176. 2325847 10.1016/0306-4522(90)90310-z

[27] Horiuchi J, McDowall LM, Dampney RA (2009) Vasomotor and respiratory responses evoked from the dorsolateral periaqueductal grey are mediated by the dorsomedial hypothalamus. J Physiol 587: 5149–5162. 19752114 10.1113/jphysiol.2009.179739 PMC2790255

[28] Xavier CH, Nalivaiko E, Beig MI, Menezes GB, Cara DC, et al (2009) Functional asymmetry in the descending cardiovascular pathways from dorsomedial hypothalamic nucleus. Neuroscience 164: 1360–1368. 19761813 10.1016/j.neuroscience.2009.09.018

[29] Xavier CH, Beig MI, Ianzer D, Fontes MA, Nalivaiko E (2013) Asymmetry in the control of cardiac performance by dorsomedial hypothalamus. Am J Physiol Regul Integr Comp Physiol 304: R664–674. 23408030 10.1152/ajpregu.00401.2012

[30] Xavier CH, Vaz GC, Lima AM, Ianzer D, Nalivaiko E, et al (2011) Contribution of excitatory amino acid receptors in the dorsomedial hypothalamus and periaqueductal gray to the asymmetry and lateralization in the cardiovascular responses to stress in rats. Autonomic Neuroscience 163: 57.

[31] Fang HS, Wang SC (1962) Cardioaccelerator and cardioaugmentor points in hypothalamus of the dog. Am J Physiol 203: 147–150.

[32] Dimicco JA, Soltis RP, Anderson JJ, Wible JH Jr (1992) Hypothalamic Mechanisms and the Cardiovascular Response to Stress. In: Kunos G, Ciriello J, editors.Central Neural Mechanisms in Cardiovascular Regulation, Vol. II. Boston, MA: Birkhauser.

[33] Soltis RP, Cook JC, Gregg AE, Stratton JM, Flickinger KA (1998) EAA receptors in the dorsomedial hypothalamic area mediate the cardiovascular response to activation of the amygdala. Am J Physiol 275: R624–631. 9688702 10.1152/ajpregu.1998.275.2.R624

[34] Myers B, Mark Dolgas C, Kasckow J, Cullinan WE, Herman JP (2013) Central stress-integrative circuits: forebrain glutamatergic and GABAergic projections to the dorsomedial hypothalamus, medial preoptic area, and bed nucleus of the stria terminalis. Brain Struct Funct 219(4): 1287–1303. 23661182 10.1007/s00429-013-0566-y PMC3778169

[35] Fontes MA, Tagawa T, Polson JW, Cavanagh SJ, Dampney RA (2001) Descending pathways mediating cardiovascular response from dorsomedial hypothalamic nucleus. Am J Physiol Heart Circ Physiol 280: H2891–2901. 11356650 10.1152/ajpheart.2001.280.6.H2891

[36] Cao WH, Fan W, Morrison SF (2004) Medullary pathways mediating specific sympathetic responses to activation of dorsomedial hypothalamus. Neuroscience 126: 229–240. 15145088 10.1016/j.neuroscience.2004.03.013

[37] Kamosinska B, Nowicki D, Szulczyk P (1989) Control of the heart rate by sympathetic nerves in cats. J Auton Nerv Syst 26: 241–249. 2754180 10.1016/0165-1838(89)90173-2

[38] Randall WC, Armour JA, Geis WP, Lippincott DB (1972) Regional cardiac distribution of the sympathetic nerves. Fed Proc 31: 1199–1208. 5038369

[39] Campos RR, McAllen RM (1999) Cardiac inotropic, chronotropic, and dromotropic actions of subretrofacial neurons of cat RVLM. Am J Physiol 276: R1102–1111. 10198391 10.1152/ajpregu.1999.276.4.R1102

[40] Yanowitz F, Preston JB, Abildskov JA (1966) Functional distribution of right and left stellate innervation to the ventricles. Production of neurogenic electrocardiographic changes by unilateral alteration of sympathetic tone. Circ Res 18: 416–428. 4952701 10.1161/01.res.18.4.416

[41] Furnival CM, Linden RJ, Snow HM (1968) Response to stimulation of the cardiac sympathetic nerves. J Physiol 197: 74P–75P. 5675089

[42] Winter J, Tanko AS, Brack KE, Coote JH, Ng GA (2012) Differential cardiac responses to unilateral sympathetic nerve stimulation in the isolated innervated rabbit heart. Auton Neurosci 166: 4–14. 21930436 10.1016/j.autneu.2011.08.004

[43] Huang J, Chowhdury SI, Weiss ML (2002) Distribution of sympathetic preganglionic neurons innervating the kidney in the rat: PRV transneuronal tracing and serial reconstruction. Auton Neurosci 95: 57–70. 11871786 10.1016/s1566-0702(01)00356-3

[44] Moon EA, Goodchild AK, Pilowsky PM (2002) Lateralisation of projections from the rostral ventrolateral medulla to sympathetic preganglionic neurons in the rat. Brain Res 929: 181–190. 11864623 10.1016/s0006-8993(01)03388-1

[45] Shafton AD, Ryan A, Badoer E (1998) Neurons in the hypothalamic paraventricular nucleus send collaterals to the spinal cord and to the rostral ventrolateral medulla in the rat. Brain Res 801: 239–243. 9729407 10.1016/s0006-8993(98)00587-3

[46] Taylor RB, Weaver LC (1992) Spinal stimulation to locate preganglionic neurons controlling the kidney, spleen, or intestine. Am J Physiol 263: H1026–1033. 1415750 10.1152/ajpheart.1992.263.4.H1026

[47] Thompson RH, Canteras NS, Swanson LW (1996) Organization of projections from the dorsomedial nucleus of the hypothalamus: a PHA-L study in the rat. J Comp Neurol 376: 143–173. 8946289 10.1002/(SICI)1096-9861(19961202)376:1<143::AID-CNE9>3.0.CO;2-3

[48] Zaretskaia MV, Zaretsky DV, Sarkar S, Shekhar A, DiMicco JA (2008) Induction of Fos-immunoreactivity in the rat brain following disinhibition of the dorsomedial hypothalamus. Brain Res 1200: 39–50. 18282559 10.1016/j.brainres.2008.01.018 PMC2323830

[49] de Oliveira RW, Del Bel EA, Guimaraes FS (2000) Behavioral and c-fos expression changes induced by nitric oxide donors microinjected into the dorsal periaqueductal gray. Brain Res Bull 51: 457–464. 10758334 10.1016/s0361-9230(99)00248-8

[50] Apps R, Ruigrok TJ (2007) A fluorescence-based double retrograde tracer strategy for charting central neuronal connections. Nat Protoc 2: 1862–1868. 17703196 10.1038/nprot.2007.263

[51] Paxinos G, Watson C (1986) The rat brain in stereotaxic coordinates. New York: Academic Press. 264 p.

[52] da Silva LG, de Menezes RC, dos Santos RA, Campagnole-Santos MJ, Fontes MA (2003) Role of periaqueductal gray on the cardiovascular response evoked by disinhibition of the dorsomedial hypothalamus. Brain Res 984: 206–214. 12932855 10.1016/s0006-8993(03)03157-3

[53] Menezes RC, Fontes MA (2007) Cardiovascular effects produced by activation of GABA receptors in the rostral ventrolateral medulla of conscious rats. Neuroscience 144: 336–343. 17049168 10.1016/j.neuroscience.2006.08.062

