# Supplementary figures and images for: Excitatory Amino Acid Receptors Mediate Asymmetry and Lateralization in the Descending Cardiovascular Pathways from the Dorsomedial Hypothalamus
Source: PLoS One. 2014 Nov 14;9(11):e112412. doi: 10.1371/journal.pone.0112412 (PMC4232378; doi:10.1371/journal.pone.0112412)

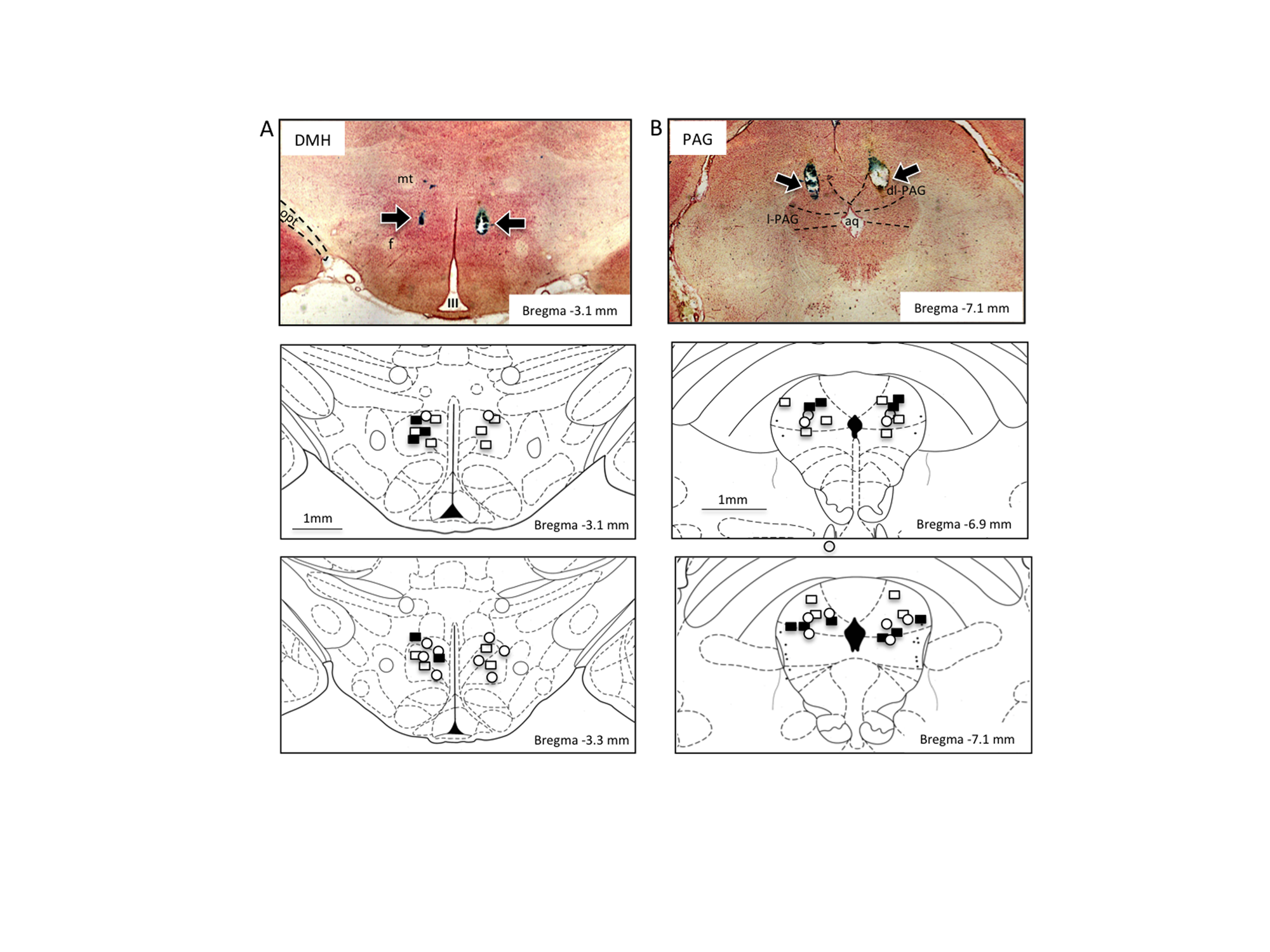

Supplement: Figure S1 — Photomicrographs of rat brain slices and schematic drawing depicting the injection sites at the level of the DMH (column A) and PAG (column B). mt: mammillothalamic tract; f: fornix; III: third ventricle; l/dlPAG: lateral/dorsolateral periaqueductal gray; Aq: aqueduct. White squares: experiment 1. Black squares: experiment 2. White circles: experiment 3. (TIF) [file pone.0112412.s001.tif]
